# *PTEN* mutation, methylation and expression in breast cancer patients

**DOI:** 10.3892/ol.2013.1331

**Published:** 2013-05-08

**Authors:** HONG-YAN ZHANG, FENG LIANG, ZHI-LING JIA, SAN-TAI SONG, ZE-FEI JIANG

**Affiliations:** 1 Department of Oncology, General Hospital of Beijing Military Area, Beijing 100700;; 2 Departments of General Surgery, Affiliated Hospital of Academy of Military Medical Sciences, Beijing 100071, P.R. China; 3 Breast Cancer, Affiliated Hospital of Academy of Military Medical Sciences, Beijing 100071, P.R. China

**Keywords:** *PTEN* gene, breast cancer, gene mutation, promoter methylation

## Abstract

The tumor suppressor gene, *PTEN*, has previously been demonstrated to be involved in breast tumorigenesis and tumor progression. The aim of the present study was to investigate the expression and significance of *PTEN* in breast carcinomas, to detect the mutation frequency of *PTEN* in sporadic breast carcinoma tissues and to determine the association between *PTEN* promoter methylation and gene expression. Immunohistochemical methods were used to analyze the expression of the *PTEN* gene in 146 cases of breast carcinoma and 10 cases of normal breast tissue closely adjacent to the carcinoma. Polymerase chain reaction-single strand conformation polymorphism (PCR-SSCP) analysis was used to analyze conformation polymorphisms in 45 breast carcinoma and 10 normal breast tissues. Point mutations of abnormal single stranded conformation were detected by DNA sequencing. The methylation of the *PTEN* promoter was analyzed by methylation-specific PCR. Expression of *PTEN* was detected in 57.5% (84/146) of patients with breast carcinoma. By contrast, *PTEN* expression was detected in 100% of normal samples. Expression of *PTEN* was found to negatively correlate with the tumor size, the pathological stage and the expression of the estrogen receptor (ER) and the progesterone receptor (PR) in breast cancer. The 2-year disease-free survival of patients with a high expression of *PTEN* was higher compared with those with low *PTEN* expression (P<0.05). Missense mutations in exon 2 of *PTEN* were identified in 1/45 breast cancer cases. *PTEN* promoter methylation was detected in 31.1% (14/45) of breast carcinomas, of which 64.3% (9/14) were associated with a loss of *PTEN* expression. The tumor suppressor gene, *PTEN*, was abnormally expressed in the breast carcinomas. The number of *PTEN* mutations were low (1/45) in the sporadic breast cancer cases analyzed in the present study and *PTEN* promoter methylation may have been the main mechanism leading to the decreased expression of *PTEN*. These results indicate that *PTEN* is important for the tumorigenesis, development and prognosis of breast cancer.

## Introduction

Breast cancer is the most common malignancy with a high mortality in females worldwide. Tumor suppressor genes are important for maintaining genome integrity and the cell cycle. *PTEN* was the first phosphatase to be identified as a tumor suppressor with diverse functions, including regulation of cell cycle, apoptosis and metastasis (1–4). Mutations or a reduced expression of the *PTEN* gene are associated with a wide variety of human tumors (5). Germline mutations in *PTEN* are known to cause Cowden syndrome (CS), which is characterized by a high risk of breast cancer. In families with CS, ∼80% have *PTEN* germline mutations and female CS patients have a 25–50% lifetime risk of developing breast cancer (6). In sporadic breast carcinomas, the frequency of *PTEN* loss is 30–40% and the somatic intragenic *PTEN* mutation frequency is <5%. Besides genetic change, aberrant DNA methylation is also responsible for the epigenetic silencing of genes associated with tumor genesis and progression of cancer. In the present study, the frequency of *PTEN* mutations, the methylation of *PTEN* and its association with the loss of *PTEN* expression were further investigated in sporadic breast carcinoma in a Chinese population.

## Material and methods

### Patients and tissue samples

A total of 146 female Chinese patients, who were diagnosed with breast cancer between 2003 and 2006, were included in the present study. Clinical and pathological information, including age, ethnicity, menopausal status, type of tumor, disease stage, axillary lymph nodes, tumor size and biomarkers, were collected. Paraffin blocks of tumor samples from all 146 patients and fresh frozen tumor specimens from 45 patients were prepared. In addition, 10 normal tissues adjacent to the tumors were also collected. All patients were followed up until December 2009. This study was approved by the ethics committee of the General Hospital of Beijing Military Area. Written informed consent was obtained from all patients.

### Sample preparation

Tissue sections (thickness, 4–5 *μ*m) were cut from the paraffin blocks for the detection of *PTEN* expression in the breast carcinoma or normal breast tissues. Genomic DNA was isolated from frozen specimens using a NucleoSpin Tissue kit (Clontech, Mountain View, CA, USA) according to the manufacturer’s instructions. DNA samples were frozen at −70°C until use.

### Immunohistochemistry

Following deparaffinization and dehydration in hydrogen peroxide, the sections were incubated at 37°C for 1 h with anti-PTEN mouse monoclonal antibody (1:25 dilution). Corresponding biotinylated anti-IgG was added and incubated for 30 min at 37°C. Next, the sections were incubated with 3,3′-diaminobenzidine (DAB) substrate chromogen solution and counterstained with hematoxylin. Negative controls were incubated with phosphate-buffered saline (PBS) instead of primary antibody. Known positive tissues were used as positive controls. Immunohistochemical reactivity was graded according to the percentage of positive tumor cells: −, 0; +, <20; ++, 20–50; and +++, >50%. Grades − or + were considered as low level expression and grades ++ or +++ were considered as high level expression.

### Analysis of PTEN gene mutation

Polymerase chain reaction-single strand conformation polymorphism (PCR-SSCP) analysis was performed to analyze mutations of the *PTEN* gene. The *PTEN* gene coding region was amplified from genomic DNA by PCR. Due to the low sensitivity of SSCP to detect sequences of >300 bp, exons 5, 8 and 9 were amplified separately. The oligonucleotide primer pairs located in exons 1–9 of the *PTEN* gene are listed in Table I. Following denaturation at 99°C for 10 min, the PCR products were chilled on ice, followed by electrophoresis on a 8% polyacrylamide gel for 12 h at 40 W. The gel was silver stained and the PCR products with aberrant bands or mobility shift were retrieved and sequenced directly.

### Analysis of DNA methylation

Methylation of the *PTEN* promoter was assessed by bisulfite treatment. This lead to chemical conversion of any unmethylated cytosine to uracil, while the methylated cytosine remained unmodified. As described previously (7), methylation specific PCR (MSP) using 2 primer pairs was designed to distinguish methylated DNA from unmethylated DNA.

### DNA modification by bisulfite treatment

The bisulfite conversion was performed using 1 *μ*g DNA. Briefly, the DNA was denatured by incubation with 10 *μ*l NaOH (1 M) for 10 min at 37°C. The samples were then treated with sodium bisulfite (3 M) and hydroquinone (10 mM) for 16 h at 55°C with salmon sperm DNA as a supporter. Modified DNA samples were purified using the Wizard DNA Purification kit (Promega, Madison, WI, USA) according to the manufacturer’s instructions. NaOH (1 M) was added and incubated for 7 min at room temperature to terminate the modification. The DNA was ethanol precipitated and dissolved in double distilled water for PCR.

### MSP

Two primer sets were used to amplify the promoter region of the *PTEN* gene, which incorporated a number of CpG sites, one specific for the methylated sequence (M, sense: 5′-TTCGTTCGTCGTCGTATTT-3′; antisense: 5′-GCCGCTTAACTCTAAACCGCAACCG-3′; PCR product, 206 bp) and the other for the unmethylated sequence (U, sense: 5′-TGTTGGTGGAGGTAGTTGTTT-3′; antisense: 5′-ACCACT TA ACTCTA A ACCACA ACCA-3′; PCR product, 162 bp) (7). The primers used in the present study specifically detect the promoter sequence of the *PTEN* gene rather than that of the *PTEN* pseudogene. The PCR volume (50 *μ*l) included 200 ng modified DNA, 20 pmol of each primer, 1.5 mmol/l MgCl_2_, 5 *μ*l PCR intensifier and 2.5 U HotStart Taq. The PCR parameters consisted of 64°C for 10 cycles, 62°C for 15 cycles and 60°C for 10 cycles, for 60 sec at each temperature. Each MSP was repeated at least 3 times.

### Statistical analysis

All comparisons between categorical variables were examined by the Fisher’s exact chi-squared test. Association analysis was performed with the Spearman’s rank correlation. Relapse-free survival was calculated using the Kaplan-Meier survival estimates and the log-rank test from the date of diagnosis until the last contact or relapse. A Cox regression analysis was performed to estimate the relative risks (with 95% confidence intervals). P<0.05 was considered to indicate a statistically significant difference. Statistical analyses were conducted using SPSS 13.0 for Windows (SPSS, Inc., Chicago, IL, USA).

## Results

### PTEN expression in breast cancer

**Figure 1.**
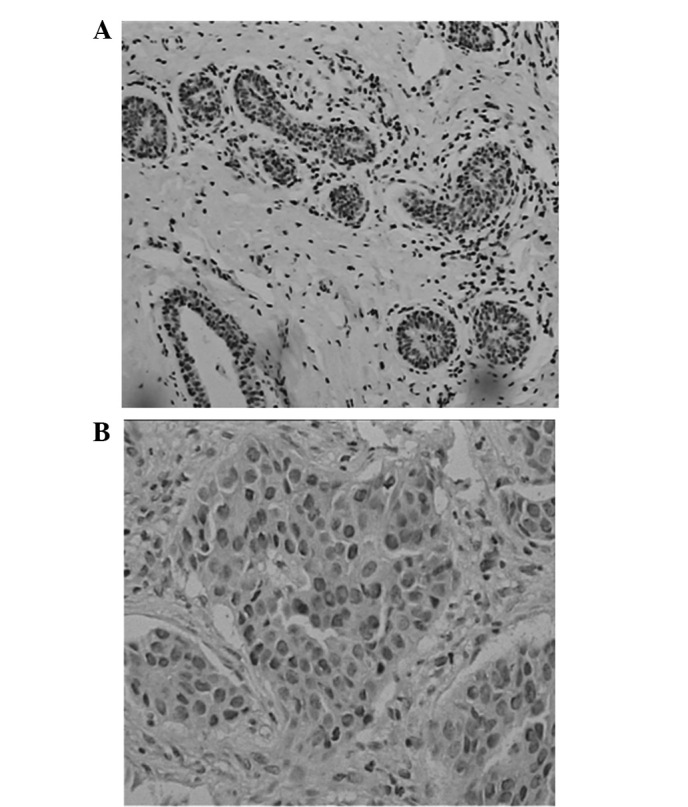
Expression of PTEN in breast tissues (A) closely adjacent to the carcinoma and (B) in the breast carcinoma.

PTEN was markedly expressed in the cytoplasm and nuclei of the breast cancer cells and in the normal duct epithelial cells (Fig. 1). PTEN-positive cells were diffusely distributed in the carcinoma. The positive expression rate of PTEN was 57.5% (84/146) in the breast cancer patients, but 100% in the normal breast tissues closely adjacent to the carcinoma (Table II).

The correlation between PTEN expression and the clinicopathological parameters, including age, disease stage, lymph node status, tumor grade, size and expression of ER, PR and Her-2/neu, was analyzed. The results revealed a negative correlation between PTEN expression and the tumor size or stage. However, no correlation was observed between PTEN expression and age, menopause or the presence of lymph node metastasis (Table II).

### PTEN expression and tumor immunophenotype

The correlation between PTEN expression and tumor immunophenotype was also analyzed and the results demonstrated that there was no correlation between PTEN expression and Her-2/neu (P=0.865). However, there was a positive correlation between the expression of PTEN and ER (P=0.023) or PR (P=0.038; Table III).

### PTEN expression and relapse-free survival

**Figure 2.**
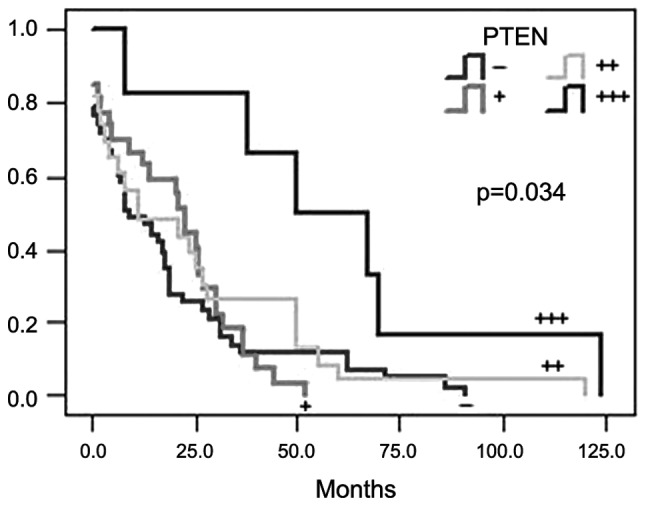
PTEN expression and relapse free survival of breast cancer; lines indicate patients with various levels of PTEN expression. The high expression of PTEN was significantly associated with a longer relapse-free survival.

**Figure 3.**
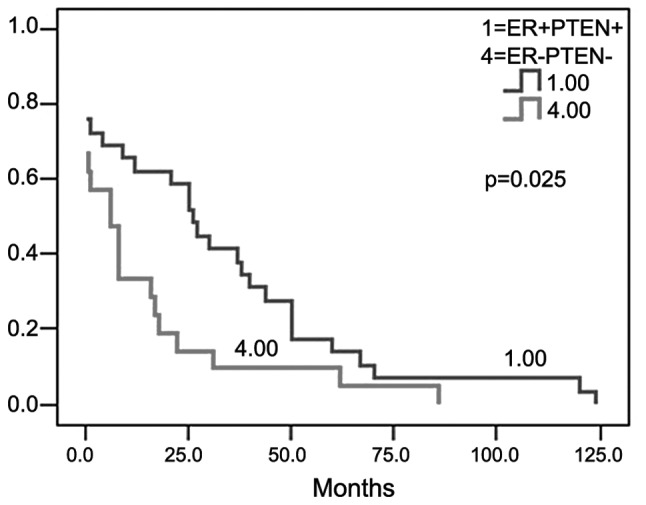
ER + PTEN expression and disease-free survival of breast cancer. The longest relapse-free survival occurred in patients that were ER and PTEN positive. ER, estrogen receptor.

Breast cancer patients (98/101) were followed up for 40–266 months (median, 103 months; 3 patients were missed). A univariate analysis revealed that the high expression of PTEN was significantly associated with a longer relapse-free survival (χ^2^=7.965; P=0.034; Fig. 2). The longest relapse-free survival occurred in patients that were ER- and PTEN-positive (χ^2^=5.044; P=0.025; Fig. 3). A Cox regression analysis demonstrated that as the prognostic factors, including menopausal status (r= 0.779; P=0.010), tumor size (r=0.836; P=0.000), lymph nodes status (r=0.806; P=0.010), adjuvant therapy (r=−0.796; P=0.016) and expression of ER (r=−0.793; P=0.018) and HER-2 (r=1.141; P= 0.002), were introduced into the equation, there was no correlation between the expression of PTEN and relapse-free survival (Table IV).

### PTEN mutation in breast cancer

**Figure 4.**
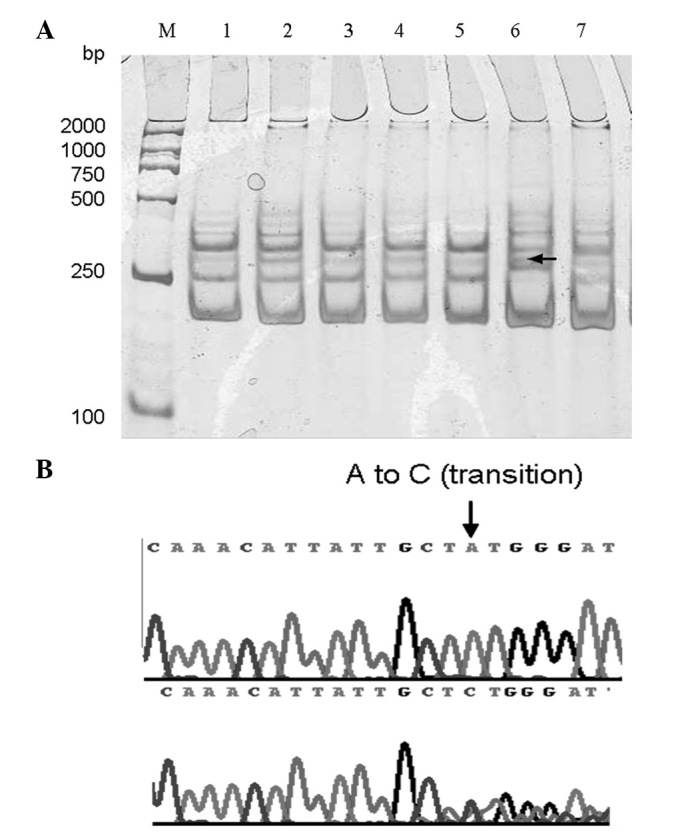
(A) Single-strand conformation polymorphism (SSCP) and sequencing analysis of PTEN mutation. Amplified DNA fragments from exons 1–9 of the PTEN gene from tumor tissues and normal tissues adjacent to the tumor. The mobility band shift was present in the polymerase chain reaction (PCR) product of the tumor DNA sample in exon 2 (arrow), indicating a conformational change in the DNA fragment (lane 6). (B) Sequence analysis of the PCR product from exon 2 of the PTEN gene. The arrow indicates a nucleotide change in codon 24, which resulted in a change from methionine to leucine. M, marker.

SSCP analysis identified no abnormal single-strand conformation in the 10 normal tissues adjacent to the tumor. Among the 45 fresh breast carcinoma samples, 3 band shifts were identified by PCR-SSCP (Fig. 4). However, only 1 mutation was confirmed in exon 2 by sequencing. The *PTEN* mutation rate was 2.2% (1/45). Sequencing analysis revealed that a codon 24 A→C missense mutation in exon 2 resulted in a codogenic amino acid change from methionine to leucine (Fig. 4).

### PTEN promoter methylation in breast cancer and its correlation with clinical manifestations

**Figure 5.**
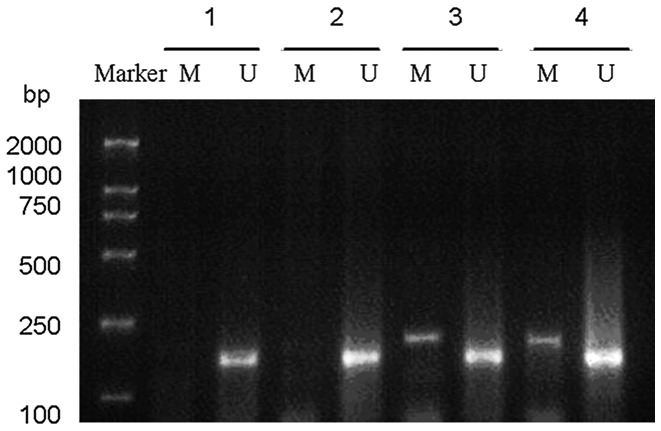
PTEN promoter CpG MSP product. Marker, 100–2000 bp. 1, normal control; 2, breast carcinoma, no PTEN methylation; 3 and 4, breast carcinoma, PTEN methylation. M, PTEN methylation; U, PTEN unmethylation; MSP, methylation specific polymerase chain reaction.

The results of the MSP analysis of *PTEN* promoter methylation in the 45 breast cancer patients are summarized in Table V and presented in Fig. 5.

The MSP analysis demonstrated that *PTEN* promoter methylation was detected in 14 breast cancer cases with a methylation rate of 31.1% (14/45; Table V and Fig. 5). Among the cases with *PTEN* promoter methylations, 64.3% (9/14) of the patients lost *PTEN* expression. The occurrence of *PTEN* promoter methylation in the patients with negative *PTEN* expression was significantly higher that in the *PTEN*-positive cases (χ^2^=4.994; P=0.025; Table V and Fig. 5).

Further clinicopathological analysis revealed that among the 14 cases with positive *PTEN* promoter methylation, 7.1% (1/14) were stage I breast cancer, 64.3% (9/14) had axillary lymph node metastasis, 42.9% (6/14) were ER-negative and 21.4% (3/14) overexpressed HER-2. However, these cases were not found to be significantly different compared with the *PTEN* methylation negative group (P>0.05).

## Discussion

*PTEN* was the first recognized tumor suppressor with lipid phosphatase activity. A previous study (3) demonstrated that *PTEN* is mutated or inactivated in a number of malignant tumors, including neuroglioma, endometrial, prostate, breast, thyroid and liver cancer. Mutation rates range between 40 and 80% in prostatic cancer, endometrial carcinoma and advanced neuroglioma, indicating that the *PTEN* mutation is important for tumorigenesis and cancer development (3). The majority of *PTEN* mutations in tumors are localized to exons 5, 7 or 8 (5,7,8). In CS, ∼40% of *PTEN* mutations are located in the exon 5 phosphatase-coding domain, leading to a reduction in its tumor suppressor activity.

In breast cancer, a 10–40% loss of heterozygosity (LOH) has been identified at the chromosome 10q23 region that contains the *PTEN* gene (6,8,9). LOH at the 10q23 region is functionally associated with the occurrence of breast cancer and this region has also been found to significantly correlate with tumor characteristics. In the study by Garcia *et al*, the breast cancer patients with LOH were younger, exhibiting a high incidence of lymph node metastases and a high histological grade (10). The loss of 10q23 is markedly associated with tumor progression (9). LOH was not observed in pure intraductal carcinomas, while it was observed in 40% (17/42) of the invasive carcinomas. LOH at 10q23 was found to correlate with the loss of ER (10).

Although LOH at 10q23 is frequent, the somatic *PTEN* mutation rate is <5% (3) and the majority of *PTEN* gene mutations occur in advanced and metastatic breast cancer. The association between *PTEN* and breast cancer is controversial and it has been hypothesized that another new gene is located in the 10q23 region. Further studies have indicated that a reduced *PTEN* expression rate occurs in 83% of breast cancer cases (11). The frequency of reduced PTEN expression in invasive cancer was higher than that in the carcinoma *in situ* (12). It was demonstrated that reduced PTEN expression was correlated with a loss of ER expression, tumor size and lymph node metastasis. Therefore, PTEN may represent an important prognostic factor.

In the present study, the expression rate of PTEN in breast carcinoma (57.5%, 84/146) was found to be significantly lower than that of normal breast tissues adjacent to tumors (100%, 10/10; P<0.05). In addition, PTEN expression was found to significantly correlate with tumor size or stage, indicating that the reduced expression of PTEN was associated with a larger tumor size or advanced stages. In addition, PTEN expression was found to correlate with ER or PR. A survival analysis revealed that the 2-year relapse-free survival rate of breast cancer patients with PTEN expression grade +++ was higher than those with lower grades of PTEN expression. The lowest rate of breast cancer relapse-free survival occurred in ER- and PTEN-negative patients. Consistent with studies by Perren *et al* (11) and Depowski *et al* (13), PTEN did not enter the equation of the COX regression analysis in the present study. By contrast, other studies have reported that PTEN is an independent prognostic factor (14,15).

PCR-SSCP and sequencing analysis detected only 1 mutation in 45 breast cancer patients and this point mutation was a missense mutation in exon 2. The patient with the *PTEN* mutation had a high risk of recurrence, infiltrating duct carcinoma, a tumor size of 1.2×2.0×2.0 cm and axillary lymph node metastasis (4/10). In addition, this patient was at the pathological stage of pT1N2M0, IIIA, ER -, PR - and Her2 ++. In the present study, the loss of PTEN expression was observed in 42.5% (62/146) of patients, while the mutation rate was only 2.2% (1/45), indicating that mechanisms other than mutation caused the loss of PTEN expression.

Shoman *et al* (15) previously demonstrated that reduced PTEN expression was associated with the shorter relapse-free survival of 100 tamoxifen-treated ER-positive breast cancer patients. When stage I patients were analyzed separately, reduced PTEN expression was a strong predictor of shorter relapse-free and disease-specific survival. When patients were stratified by levels of ER expression (≥50% vs. <50% positive cells), reduced PTEN expression was associated with a less favorable outcome in each patient group. Among the tumor patients with a normal expression of PTEN, 30% relapsed and 25% succumbed to their condition. By contrast, among the tumor patients with a reduced expression of PTEN, 90% relapsed and 65% succumbed to their condition. However, in this earlier study, ER-negative breast cancer cases and patients not treated with tamoxifen were not included. This study and the results of the present study indicate a correlation between PTEN and ER, and the combination of PTEN and ER may predict an outcome for breast cancer patients.

Estrogen-induced proliferation of mammary and uterine epithelial cells is primarily mediated by ER via estrogen-independent (AF-1) and estrogen-dependent (AF-2) activation domains. These domains regulate gene transcription by recruiting co-activators and interacting with the general transcriptional machinery. *In vitro* studies have demonstrated that phosphatidylinositol 3-kinase (PI3K) and AKT activate ER in the absence of estrogen (16). PI3K was shown to increase the activities of the AF-1 and AF-2 domains of ER, while AKT increased the activity of the AF-1 domain only. PTEN and a catalytically inactive AKT are able to downregulate PI3K-induced AF-1 activity, indicating that PI3K activates ER via AKT-dependent and -independent pathways. It has been demonstrated that the activation of the PI3K/AKT survival pathways and the hormone-independent activation of ER are associated with the inhibition of tamoxifen-induced apoptotic regression. AKT protects breast cancer cells from tamoxifen-induced apoptosis. The downregulation of PTEN expression results in a loss of the inhibition of PI3K/AKT. By contrast, the high expression of PTEN improves the response of breast cancer to tamoxifen. Therefore, breast cancer patients with a positive expression of PTEN and ER are associated with longer survival (16).

In addition to gene mutation, epigenetic regulation, including promoter hypermethylation, has been demonstrated to alter tumor suppressor gene expression and contribute to tumorigenesis. Thus, the methylation status of the *PTEN* promoter CpG island was analyzed in the present study. As one of the most recurrent gene alterations, DNA methylation significantly affects chromosomal formation, gene expression and DNA replication (17,18). CpG islands in the promoter or nearby regions are frequently methylated, leading to the silencing of gene transcription. Methylation of a growing number of tumor suppressor genes, including p16, APC, MLH1 and BRCA1, has been revealed to be one of the most frequent mechanisms of gene transcription inactivation and loss of gene function. Soria *et al* (19) analyzed the methylation of the *PTEN* promoter and the *PTEN* expression in 30 cases of non-small cell lung cancer (NSCLC). The results indicated that *PTEN* methylation occurred in 35% (7/20) of NSCLC cases and 69% (11/16) of NSCLC cell lines. In addition, *PTEN* methylation was not detected in patients with positive PTEN expression. Thus, the loss of PTEN expression was reported to correlate with the methylation of its promoter. *PTEN* methylation was considered as one of main causes of loss of PTEN expression. In the present study, *PTEN* methylation was detected in 31.1% (14/45) of breast cancer cases. Among these, 14 tissues with *PTEN* methylation, 64.3% (9/14), exhibited a loss of PTEN expression. The *PTEN* promoter methylation rate was 50% (9/18) in the PTEN-negative cases, which is statistically different from the rate in the PTEN-positive cases (18.5%, 5/27; P=0.025). Therefore, none of the normal breast tissues adjacent to the tumor were found to exhibit methylated *PTEN* promoters. These results are consistent with a previous study (20) demonstrating that *PTEN* methylation occurred in 34% of cases of breast invasive ductal cancer and that 60% of these were found to exhibit a loss of PTEN expression. In addition, none of the breast cancer cell lines and normal breast tissues were observed to have *PTEN* methylation. These results indicated that the methylation of the *PTEN* promoter may result in *PTEN* inactivation in breast cancer.

Previous studies have demonstrated that the PI3K/AKT/PTEN pathway is important for tumorigenesis. The PI3K signaling pathway is associated with almost all aspects of tumor biology, including cell transformation, growth, proliferation, migration and apoptosis evasion and genomic instability, angiogenesis, metastasis and cancer stem cell maintenance (21,22). PTEN degrades the product PI3K by dephosphorylating phosphatidylinositol 3,4,5-trisphosphate and phosphatidylinositol 3,4-bisphosphate at the 3’ position (23). The loss of function or reduced expression of PTEN leads to the accumulation of critical messenger lipids, which in turn increases AKT phosphorylation and activity, leading to decreased apoptosis and/or increased mitogen signaling (24–28). However, although the PI3K/AKT/PTEN pathway contains a number of attractive therapeutic targets, clinical trials of pathway-targeted drugs have not proved as promising as expected. It is possible that a novel signaling pathway playing a significant role in the PI3K/AKT/PTEN pathway has yet to be identified or that present markers are not sufficient to assess therapeutic response (29–30).

## Figures and Tables

**Table I.** PCR primers designed for 9 PTEN exons and MSP of PTEN

Primer	Sense (5′-3′)	Antisense (5′-3′)	bp
Exon			
1	TTCTGCCATCTCTCTCCTCC	ATCCGTCTACTCCCACGTTC	194
2	GTTTGATTGCTGCATATTTCA	TCTAAATGAAAACACAACATGAA	201
3	AGCTCATTTTTGTTAATGGTGG	CCTCACTCTAACAAGCAGATAACTTTC	178
4	AAAGATTCAGGCAATGTTTGTTAG	TGACAGTAAGATACAGTCTATCGGG	200
5-1	TTTTTTCTTATTCTGAGGTTATC	TCATTACACCAGTTCGTCC	184
5-2	TCATGTTGCAGCAATTCAC	GAAGAGGAAAGGAAAAACATC	176
6	ATGGCTACGACCCAGTTACC	AAGAAAACTGTTCCAATACATGG	284
7	CAGTTAAAGGCATTTCCTGTG	GCTTTTAATCTGTCCTTATTTTGG	274
8-1	TTAACATAGGTGACAGATTTTC	CACGCTCTATACTGCAAATG	222
8-2	CATTCTTCATACCAGGACCAG	TGGAGAAAAGTATCGGTTGG	188
8-3	GCATTTGCAGTATAGAGCGTG	TCAAGCAAGTTCTTCATCAGC	217
9-1	AGATGAGTCATATTTGTGGG	CTCTGGATCAGAGTCAGTGG	185
9-2	AATCCAGAGGCTAGCAGTTC	AAGGTCCATTTTCAGTTTATTC	213
MSP			
Methylated	TTCGTTCGTCGTCGTATTT	GCCGCTTAACTCTAAACCGCAACCG	206
Unmethylated	GTGTTGGTGGAGGTAGTTGTTT	ACCACTTAACTCTAAACCACAACCA	162

MSP, methylation-specific polymerase chain reaction.

**Table II.** Correlation between PTEN expression and breast cancer pathological characteristics.

Factors	n	PTEN								P-value
		−		+		++		+++		
		n	%	n	%	n	%	n	%	
Age (years)	146									
<35		14	56.0	5	20.0	5	20.0	1	4.0	0.150
35–55		38	40.0	27	28.4	18	18.9	12	12.6	
>55		10	38.5	4	15.4	11	42.3	1	3.8	
Menopause	146									
No		46	47.4	22	22.7	19	19.6	10	10.3	0.162
Yes		16	32.7	14	28.6	15	30.6	4	8.2	
T-stage	146									
T1		8	22.2	11	30.6	10	27.8	7	19.4	0.002
T2		34	47.2	15	20.8	17	23.6	6	8.3	
T3		12	52.2	5	21.7	5	21.7	1	4.3	
T4		8	53.3	5	33.3	2	13.3	0	0.0	
Lymph node metastasis	146									
No		18	38.3	11	23.4	12	25.5	6	12.8	0.630
Yes		45	45.5	25	25.3	22	22.2	7	7.1	
Stage	146									
I		3	20.0	4	26.7	4	26.7	4	26.7	0.005
II		25	39.1	16	25.0	16	25.0	7	10.9	
III		28	47.5	15	25.4	13	22.0	3	5.1	
IV		6	75.0	1	12.5	1	12.5	0	0.0	

Immunohistochemical reactivity graded according to the percentage of positive tumor cells:

−, 0;

+, <20;

++, 20–50; and

+++, >50%.

**Table III.** PTEN expression and tumor immunophenotype (Spearmen’s rank correlation)

Gene	PTEN								χ^2^	P-value
	−		+		++		+++			
	n	%	n	%	n	%	n	%		
ER										
−	21	42.9	16	32.7	12	24.5	0	0	2.303	0.023
+	32	48.5	15	22.7	12	18.2	7	10.6		
++	7	35.0	3	15.0	6	30.0	4	20.0		
+++	2	18.2	2	18.2	4	36.4	3	27.3		
PR										
−	29	48.3	15	25.0	11	18.3	5	8.3	2.091	0.038
+	26	41.9	17	27.4	15	24.2	4	6.5		
++	7	36.8	4	21.1	5	26.3	3	15.8		
+++	0	0.0	0	0.0	3	60.0	2	40.0		
HER-2										
−	34	46.6	15	20.5	16	21.9	8	11.0	0.081	0.865
+	11	32.4	8	23.5	9	26.5	6	17.6		
++	10	37.0	12	44.4	5	18.5	0	0.0		
+++	7	58.3	1	8.3	4	33.3	0	0.0		

Immunohistochemical reactivity graded according to the percentage of positive tumor cells:

−, 0;

+, <20;

++, 20–50; and

+++, >50%. ER, estrogen receptor; PR, progesterone receptor.

**Table IV.** Cox regression analysis estimated prognostic value by hazard ratios and 95% CI for relapse-free survival

Variable	B	SE	Wald	P-value	Exp (B)	95% CI for Exp (B)	
						Lower	Upper
Age	0.190	0.348	0.298	0.585	1.209	0.611	2.393
Menopause	0.779	0.303	6.596	0.010	2.180	1.203	3.951
Tumor size	0.836	0.202	17.077	0.000	2.306	1.552	3.428
Lymph node	0.806	0.312	6.673	0.010	2.238	1.215	4.125
Adjunct therapy	−0.796	0.332	5.757	0.016	0.451	0.235	0.864
ER	−0.793	0.334	5.636	0.018	0.453	0.235	0.871
PR	0.440	0.322	1.861	0.173	1.552	0.825	2.918
HER	−1.141	0.373	9.328	0.002	0.320	0.154	0.665
PTEN	−0.241	0.142	2.893	0.089	0.786	0.595	1.037

B, partial regression coefficent; SE, standard error; Wald, statistical magnitude; Exp (B), relative risk; CI, confidence interval; ER, estrogen receptor; PR, progesterone receptor.

**Table V.** Clinicopathological features, PTEN expression and promoter methylation in breast carcinoma.

Groups	Total cases (n)	Methylation of PTEN		χ^2^	P-value
		n	%		
PTEN expression					
−	18	9	50.0	4.994	0.025
+	27	5	18.5		
Lymph node					
−	18	5	27.8	0.733	0.392
+	27	9	33.3		
Stage					
I	7	1	14.3	1.425	0.490
II	21	8	38.1		
III	17	5	29.4		
T stage					
T1	15	3	20.0	2.118	0.347
T2	24	10	41.7		
T3	6	1	16.7		
ER					
−	25	6	24.0	0.249	1.327
+	20	8	40.0		
HER-2 expression					
Low	33	11	33.3	0.285	0.593
High	12	3	25.0		

+, positive;

−, negative;

ER, estrogen receptor.
